# 

**Published:** 1855-12

**Authors:** 


					﻿Will soon be published, from the press of Messrs. Blanchard & Lea, of
Philadelphia, “The Principles and Practice of Physical Exploration, as ap-
plied to the Diagnosis of Diseases of the Respiratory Organs. By Austin
Flint, M. D.”
Octavo of from 400 to 500 pages.
The well known ability of the writer is a sufficient guarantee that this
work will be a valuable contribution to that department of medical research.
We hope that it may meet with a warm reception among the author’s old
friends, the subscribers of the Buffalo Medical Journal.
To our Readers in Michigan.—Mr. Frederick Stearns, for a long time
connected with the firm of A. I. Mathews & Co., Druggists in this city, is
about to establish himself in Detroit as a partner in the firm of Higby &
Stearns.
We understand that the new firm, like the Buffalo house alluded to, will
refrain from the sale of patent medicines, and looking to the patronage of
the profession for support, will offer for sale only the purest and best
medicines.
Mr. Stearns, by a long course of honorable conduct here, has made many
friends. He has also the xnerit of being a skillful pharmaceutist, educated
to, and thoroughly possessed of, the minutite of his business. We commend
him to our numerous friends in Michigan, as reliable and worthy of their
support.
Boohs Received.—The “Transactions of the American Medical Associa-
tion,” “Transactions of the State Medical Society of Pennsylvania,” “Ne-
laton’s Clinical Lectures on Surgery,” “Simpson’s Obstetric Works,” and
“Beasley’s Prescription Book,” are on our table and shall have speedy
notice.
To Correspondents.—We have received several articles for publication,
which we shall endeavor to publish in our next. We must beg the patience
of our friends. Our eclectic department has dwindled to nothing under the
pressure of original matter, and we find ourself lacking elbow-room for edi-
torials. We have given up all hopes of ever editing our Journal with a pair
of scissors.
				

